# What matters to short interpregnancy intervals between pregnancies in Western Australia?

**DOI:** 10.23889/ijpds.v7i3.2029

**Published:** 2022-08-25

**Authors:** Gizachew Tessema, Jennifer Dunne, Gavin Pereira

**Affiliations:** 1 Curtin University

## Objectives

Studies showed that short interpregnancy interval - time between birth of the previous (index) child to the conception of the next - is associated with adverse perinatal and maternal outcomes, impacting sibling relations, parenting, and couple relations. This study aims to investigate the demographic and obstetric determinants of short interpregnancy interval in Western Australia.

## Approach

We used a longitudinal study using data linkage obtained from Western Australian Midwives Notification System and Hospital Morbidity Data System for 150,712 women who gave births between 2006 and 2010. We estimated unadjusted and adjusted hazard ratio (aHR) and 95% CI using Gompertz gamma shared frailty model. In the study, event was deemed when women conceived within 18 months after index (previous) births. Otherwise, we considered as censored when women conceived after 18 months of the index births or if they did not conceive until the last date of the follow up period (31st December 2015).

## Results

We found that 20% (n=30,916) of births in WA conceived within 18 months after the index pregnancy. We found that women aged <20 years (aHR=1.46, 95% CI: 1.37, 1.54), 20-34 years (aHR=1.75, 95% CI: 1.69, 1.81), women with stillbirths (HR=3.31, 95% CI: 3.00, 3.65) and women in the lowest socioeconomic status (HR=1.14, 95% CI: 1.10, 1.19) had a greater hazard of having short interpregnancy intervals. However, women with previous gestational diabetes mellitus (aHR=0,90, 95% CI: 0.85, 0.95), women with previous preeclampsia (aHR=0,92 95% CI: 0.87, 0.90), women with more than one previous birth ((aHR=0,41; 95% CI: 0.40, 0.42), women with multiple births (aHR=0,65, 95% CI: 0.60, 0.71) had less hazards than their counterparts to have short interpregnancy intervals.

## Conclusion

One in five Western Australian women conceived within the sub-optimal range of interpregnancy interval. Demographic and obstetric factors play a significant role in determining short interpregnancy intervals.

